# Reliability and reproducibility of measurements in para-sagittal planes on sub-axial cervical vertebral bodies: a morphometric study of endplates in three-dimensional models

**DOI:** 10.1186/s13018-021-02648-3

**Published:** 2021-08-16

**Authors:** Long Wang, Hao T. Luo, Wei Lu, Xing Bo Cai, Chen Yu, Sheng Lu

**Affiliations:** 1 Postgraduate College of Kunming Medical University, No.1168 Chunrong Xi Road, Chenggong District, Kunming, China; 2 Department of Orthopaedics, The First People’s Hospital of Yunnan Province, No.157, Jinbi Rd, Kunming, Yunnan China; 3 Key Laboratory of Digital Orthopedics of Yunnan Province, No.157, Jinbi Rd, Kunming, Yunnan China

**Keywords:** Cervical endplate morphology, 3D model, Para-sagittal planes, Dimension measurement

## Abstract

**Background:**

Dimensional measurements have been implemented on a variety of entities in morphological studies of the sub-axial cervical vertebral endplate. Despite great progress, little information between the mid-sagittal plane and bilateral uncinate processes has been acquired due to the lack of a reliable method to determine the para-sagittal planes. Also, few studies of this region are available. We proposed a new approach to defining the para-sagittal planes on a 3D cervical vertebral body model; in this approach, dimensions can be measured in a specific plane. The aim of this study was to assess the inter-observer and intra-observer reliability of the measurements in different sagittal planes on sub-axial cervical vertebral endplates of 3D models.

**Methods:**

We established mid-sagittal and bilateral quarter para-sagittal planes on the 3D model of a sub-axial cervical vertebral body based on landmarks labeled on the surface. By intersecting the vertebral body with the planes, three curves located at the three para-sagittal planes were generated. Linear dimensions were measured on every curve by two observers separately, and in total, 24 sub-axial cervical spines were included in the study. The first observer (O1) performed the procedure twice with an interval of 2 weeks. The paired *t* test, Wilcoxon matched-pairs signed-rank test and the interclass correlation coefficient (ICC) were employed to evaluate the inter- and intra-observer reliability of the proposed method.

**Results:**

There were no significant differences in most intra- and inter-observer comparisons, and higher non-significant proportions were found in the intra-observer comparisons than in comparisons between different observers. The interclass correlation coefficients (ICCs) in the measurements were excellent (> 0.75) in most circumstances, and the values in intra-observer comparisons were higher than those in inter-observer comparisons.

**Conclusions:**

In this study, we proposed an approach to determine the bilateral quarter para-sagittal planes in a 3D cervical vertebral body model; the results demonstrated that the method is reproducible with high intra- and inter-observer agreement.

## Background

Morphometric studies of sub-axial cervical endplates have increased over the past decades [1], and the main purpose is to provide anatomic data for the design or optimization of the intervertebral instrument and preoperative planning. Dimensional measurements of the endplates have been carried out over a series of entities, from cadaveric vertebrae and radiographic images in the early days of implementing this approach [2, 3] and have progressed to include technically advanced methods such as CT and MRI in recent years [4, 5]. Also, as a newly developed technique in morphological studies, digitized three-dimensional (3D) models have become more and more popular since their emergence [5–8]. Among the dimensions measured in these studies, depth and width are the most frequently implemented, angular dimensions were common in many studies, and the radius or diameter of an arc could be easily calculated by fitting a curve with the assistance of computer programs [9].

Although significant progress has been made in medical imaging, image segmentation, 3D model generation, and measuring techniques, further geometric knowledge remains to be elucidated at the region between the mid-sagittal plane (MSP) and bilateral uncinate processes (UPs), and a limited number of studies are available. One possible explanation is that it is not easy to accurately determine these regions on cadaveric vertebral bodies due to the few identifiable anatomic landmarks available. Panjabi et al. made para-sagittal sections of cadaveric vertebral bodies in increments of 5 mm and used a cutting machine to measure the thickness of the cortical bone of cervical vertebral bodies [10]. In another study by Ebraheim et al., bilateral para-sagittal planes were defined on a cervical vertebra 5 mm lateral to the mid-sagittal plane to evaluate the safety of anterior screw placement [11]. Feng et al. defined the curves on para-sagittal planes in a 3D model generated from optical scans of a cadaveric vertebral endplate [5] in a recent study.

In this study, we proposed an approach to define the bilateral quarter para-sagittal planes on a 3D sub-axial cervical vertebra model based on landmarks labelled on the anatomic features of the vertebral bodies; measurements can be carried out on these planes. The series of dimensions can better represent the shape of the endplates. The objective of the study was to assess the reliability and reproducibility of the method between two different observers.

## Method

### Data collection

Twenty-four CT scans of the sub-axial cervical spine were included in this study after we retrospectively reviewed the database of the hospital. All CT data were collected under the same scanning standard (slice thickness 0.625 mm, LightSpeed VCT, GE medical system, USA). Evaluated patients included 12 males, with a mean age of 34.5 ± 14.8 years (18–61 years), and 12 females, aged 40.2 ± 14.7 years (18–63 years). All CT data were selected by two senior residents specializing in spine surgery and subsequently reviewed by one specialist in the same subject. The inclusion criteria mainly included images of the adult cervical spine. Participants with pathological conditions of the cervical spine, such as severe degeneration, deformities, tumor, trauma, and previous surgeries were excluded. All participants in the study were informed of the research and approved the use of their data in this study. All patients from whom CT data were acquired were anonymized. The institutional board of review approved this study.

### Segmentation and 3D model reconstruction

**Fig. 1:**
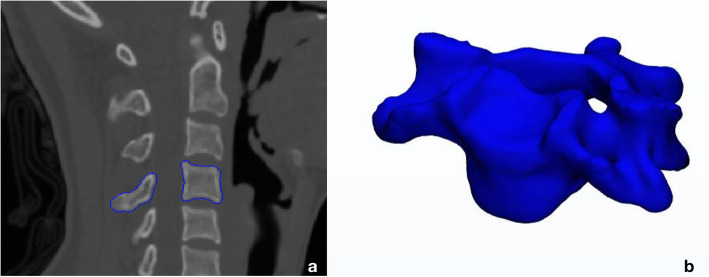
Segmentation of the C4 vertebra (**a**) and the 3D surface model generated (**b**)

The CT data were segmented based on the CT value (> 226 HU), and a 3D surface model of every sub-axial vertebra was generated (Mimics 19.0, Materialise, Leuven, Belgium) (Fig. 1).

### Reference plane establishment and curve generation

**Fig. 2:**
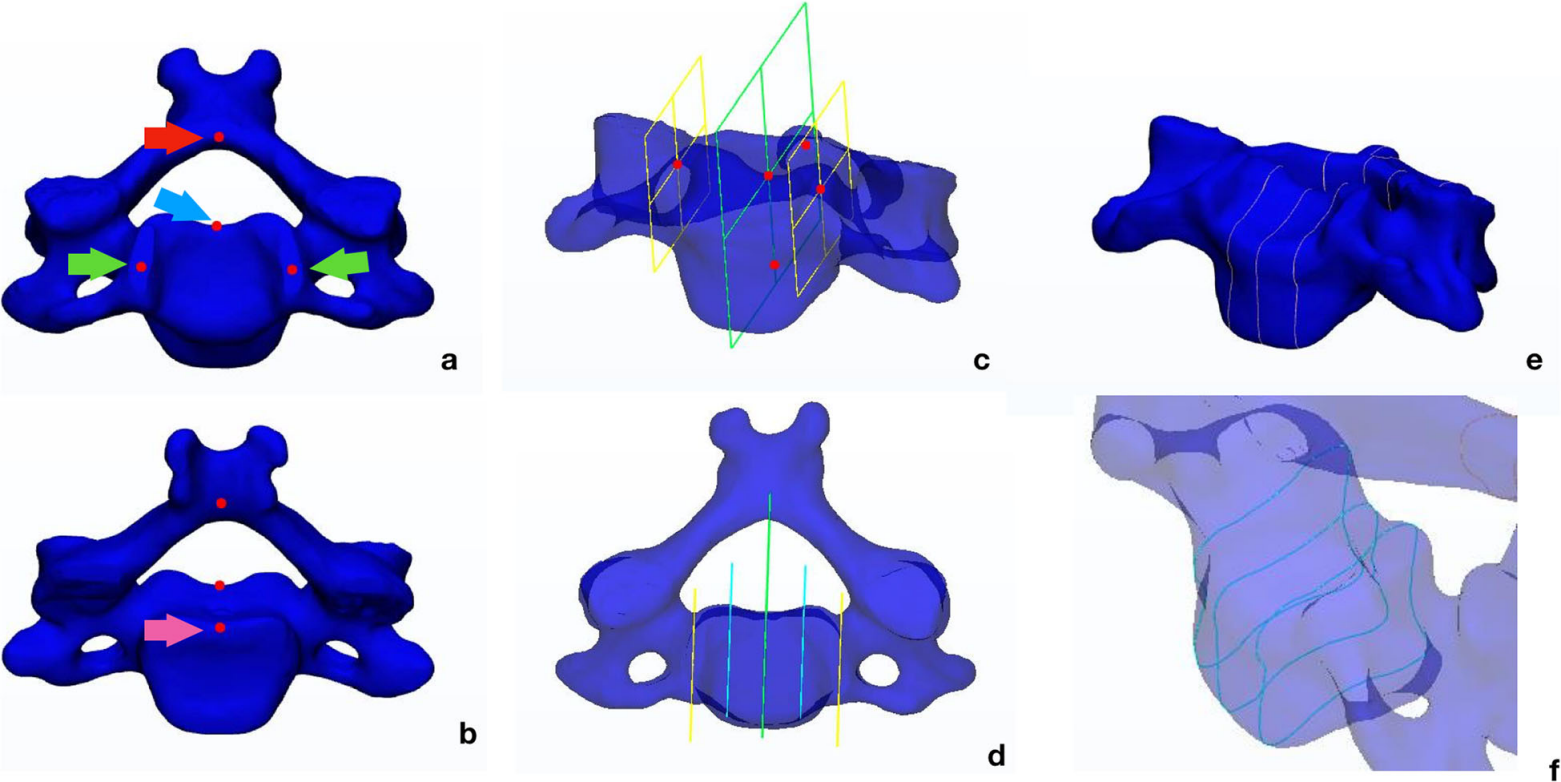
Curve generation from a 3D model. The landmarks are labeled as follows: the posterior endplate valley (PEV, the blue arrow), the intersection of two laminae (red arrow), the lateral tips of the bilateral uncinate processes (green arrows), and the midpoint of the posterior rim of the inferior endplate (pink arrow) (**a**, **b**). The reference planes that were generated are shown (**c**, **d**). The result of curve generation performed by intersecting the middle three reference planes with the vertebral body is shown (**e**, **f**)

After importing the 3D surface model into the engineering software 3-Matics 11.0 (Materialise, Leuven, Belgium), we generated the mid-sagittal plane (MSP) of the vertebral body through landmarks labelled on the vertebral body and the intersection of the bilateral laminae (Fig. 2). After that, two lateral para-sagittal planes were built through the landmarks positioned on the tips of the uncinate processes (UPs) of both sides (Fig. 2). Last, two quarter para-sagittal planes (QPSPs) of the vertebral body were calculated as the average planes between the mid-sagittal plane and the lateral planes (Fig. 2).

By intersecting the vertebral body with the three planes, the mid-sagittal plane and two quarter para-sagittal planes, three closed curves were generated (Fig. 2).

### Measurement

**Fig. 3:**
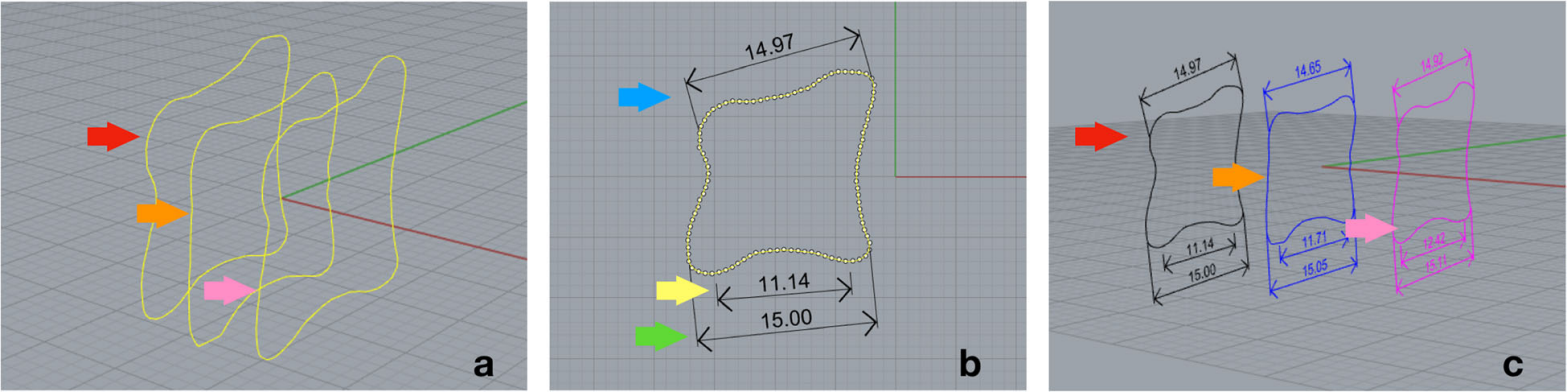
Measurements in the three planes. Re-alignment in the coordinate system was performed in the following order: right quarter para-sagittal plane (red arrow), mid-sagittal plane, and left quarter para-sagittal plane (**a**). Measurements of the curve on the right quarter para-sagittal plane, depth of the superior endplate (RSED, blue arrow), depth of the inferior endplate concave (RIECD, yellow arrow), depth of the inferior depth (RIED, green arrow) (**b**). Spatial display of the three sagittal planes with measurements (**c**)

We then exported the three curves into the 3D modelling software Rhinoceros 6.0 (Rhinoceros, Robert McNeel and Associates, Seattle, Washington, USA) for further assessment. After re-alignment in the coordinate system, the three curves were evaluated separately (Fig. 3). Three linear dimensions on every single curve were obtained, including the anteroposterior depth of the superior (SED) and the inferior endplate (IED), according to the manner proposed by Panjabi et al. [10]; the depth of inferior endplate concave (IECD) (Fig. 3); and the distance between anterior and posterior rims of the inferior endplate concave [12].

Two observers, including one musculoskeletal digitization engineer (observer one, O1) and one spine surgeon in fellowship training (observer two, O2), both of whom were experienced in 3D modelling software and were familiar with the procedures. The two observers independently performed modelling assessments and measurements of all enrolled sub-axial cervical vertebrae (C3–C7), and one of them (observer one, O1) repeated the modelling and measurements twice with an assessment interval of two weeks.

### Statistical analysis

The statistical software packages SPSS 18.0 (SPSS Inc., Chicago, IL, USA) and Prism 8.0 (GraphPad, USA) were employed to analyze the data and draw the graphs. Descriptive statistics was used for general data analysis, and the paired *t* test and the Wilcoxon matched-pairs signed-rank test were used for the comparison of the measurements. Interclass correlation coefficients (ICCs) were applied for the inter-observer and intra-observer reliability evaluation; *p* < 0.05 was considered to indicate statistical significance.

## Result

**Fig. 4:**
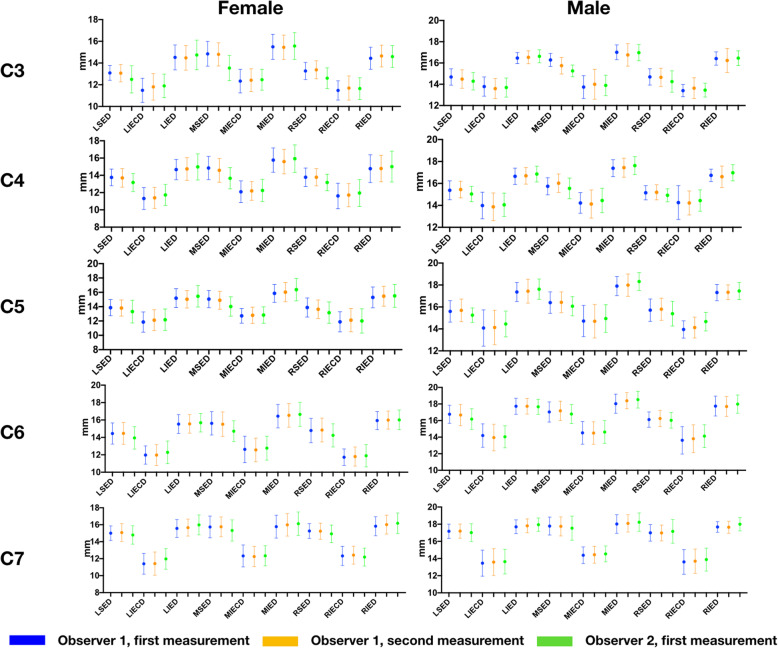
Measurement values in different planes in C3–7 of females and males (mean ± SD). LSED, depth of the superior endplate of the left para-sagittal plane. LIECD, depth of the inferior endplate concave of the left para-sagittal plane. LIED, depth of the superior endplate of the left para-sagittal plane. MSED, depth of the superior endplate of the mid-sagittal plane. MIECD, depth of the inferior endplate concave of the mid-sagittal plane. MIED, depth of the superior endplate of the mid-sagittal plane. RSED, depth of the superior endplate of the right para-sagittal plane. RIECD, depth of the inferior endplate concave of the right para-sagittal plane. RIED, depth of the superior endplate of the right para-sagittal plane

The values measured in males were greater than those in females and increased from C3 to C7 in almost all dimensions, (Fig. 4), and the trends of variations between men and women were similar. The discrepancies between the depth of the superior endplate (SED) and depth of the inferior endplate (IED) declined from C3 to C7 in the three sagittal planes (Fig. 4). The values measured on mid-sagittal planes and trends were consistent with the other studies of the measurement of the sub-axial cervical vertebra [1, 4, 7]. There were no significant differences between the values measured by the two observers and observer one (O1).

For the comparisons between values measured by the two independent observers and measured by O1 at different times, a paired *t* test was employed, and for measurement values that did not fit the normality distribution (Shapiro-Wilk test), Wilcoxon matched-pairs signed-rank test was employed. No significant differences existed in most comparisons, and there were higher non-significant proportions in the intra-observer comparisons compared with the comparisons between different observers (Tables 1 and 2).

**Table 1:** Comparison between different measurements in female

	LSED		LIECD		LIED		MSED		MIECD		MIED		RSED		RIECD		RIED	
	Intra	Inter	Intra	Inter	Intra	Inter	Intra	Inter	Intra	Inter	Intra	Inter	Intra	Inter	Intra	Inter	Intra	Inter
**C3**	0.72	0.03	0.05	0.02	0.45*	0.18	0.30	< 0.01	0.24	0.10	0.39	0.40	0.08	< 0.01	0.13	0.28	< 0.01*	0.29*
**C4**	0.45	0.01	0.64	< 0.01	0.74	0.13	0.01	< 0.01	0.23	0.17	0.03	0.27	0.98	< 0.01	0.43	0.1	0.75	0.17
**C5**	0.63	0.04	0.13	0.09	0.59	0.08	0.10	< 0.01	0.55	0.54	0.40	0.06	0.42	< 0.01	0.15*	0.60	0.02	0.20
**C6**	0.79	< 0.01	0.97	0.17	0.41	0.16	0.28	< 0.01	0.71	0.34	0.50	0.10	0.33	< 0.01	0.31	0.40	0.37	0.50
**C7**	0.37	0.12	0.87	0.01	0.06	0.02	0.65*	0.02	0.53	0.92	0.06	0.08	0.77	0.02	0.12	0.40	0.06	0.03

*Wilcoxon matched-pairs signed-rank test

*LSED* depth of the superior endplate of the left para-sagittal plane, *LIECD* depth of the inferior endplate concave of the left para-sagittal plane, *LIED* depth of the superior endplate of the left para-sagittal plane, *MSED* depth of the superior endplate of the mid-sagittal plane, *MIECD* depth of the inferior endplate concave of the mid-sagittal plane, *MIED* depth of the superior endplate of the mid-sagittal plane, *RSED* depth of the superior endplate of the right para-sagittal plane, *RIECD* depth of the inferior endplate concave of the right para-sagittal plane, *RIED* depth of the superior endplate of the right para-sagittal plane

**Table 2:** Comparison between different measurements in male

	LSED		LIECD		LIED		MSED		MIECD		MIED		RSED		RIECD		RIED	
	Intra	Inter	Intra	Inter	Intra	Inter	Intra	Inter	Intra	Inter	Intra	Inter	Intra	Inter	Intra	Inter	Intra	Inter
**C3**	0.07	< 0.01*	0.23	0.60	0.44	0.13*	0.02	< 0.01*	0.31	0.16	0.37*	0.66	0.69	0.04	0.50*	0.64	0.92*	0.61
**C4**	0.56*	0.05*	0.26	0.75	0.60	0.04	0.04	0.03	0.63	0.26	0.85	< 0.01*	0.77	0.20	0.84*	0.24*	0.73*	0.03
**C5**	0.41	0.04	0.75	0.34*	0.43	0.04	0.78	0.02	0.86	0.26	0.29	0.01	0.40	0.01	0.07	< 0.01	0.73*	0.12*
**C6**	0.50	0.12	0.20	0.55	0.98	0.56	0.19	0.05	0.68	0.51	0.08	0.02	0.10	0.40	0.40	0.39	0.18	0.10
**C7**	0.76	0.31	0.02	0.55	0.02	0.01	0.70	0.06	0.28	0.50	0.21	0.06	0.80	0.38	0.64	0.07	0.65	< 0.01

* Wilcoxon matched-pairs signed-rank test

*LSED* depth of the superior endplate of the left para-sagittal plane, *LIECD* depth of the inferior endplate concave of the left para-sagittal plane, *LIED* depth of the superior endplate of the left para-sagittal plane, *MSED* depth of the superior endplate of the mid-sagittal plane, *MIECD* depth of the inferior endplate concave of the mid-sagittal plane, *MIED* depth of the superior endplate of the mid-sagittal plane, *RSED* depth of the superior endplate of the right para-sagittal plane, *RIECD* depth of the inferior endplate concave of the right para-sagittal plane, *RIED* depth of the superior endplate of the right para-sagittal plane

**Fig. 5:**
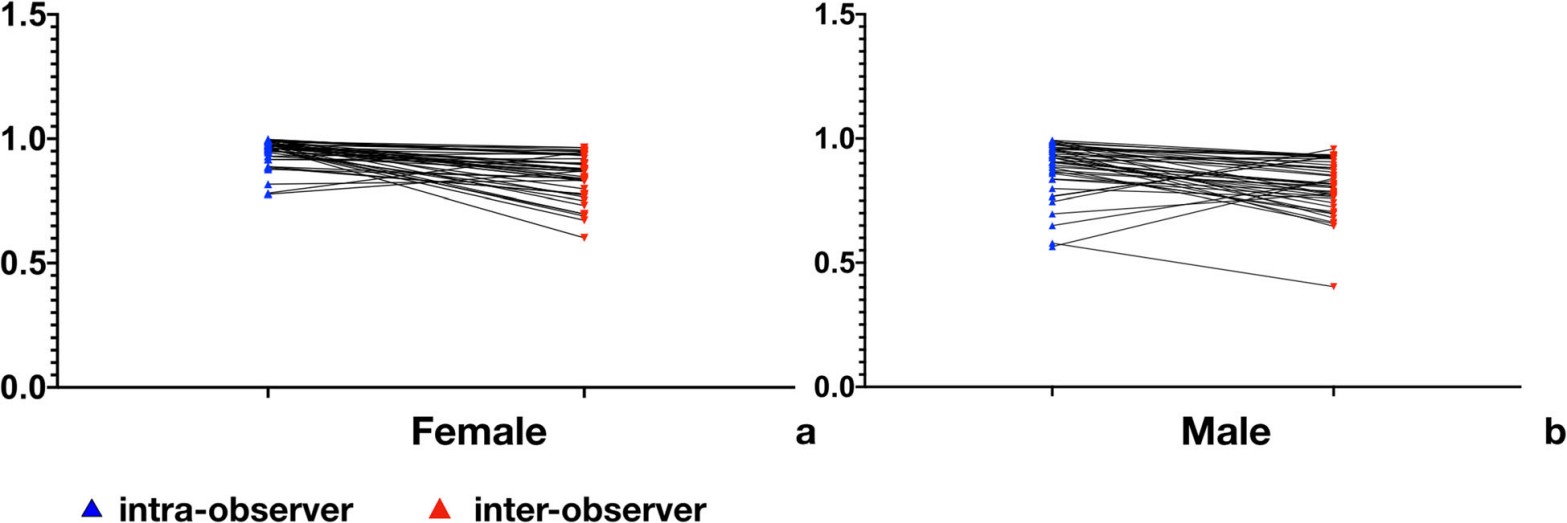
Intra-observer and inter-observer interclass correlation coefficients (ICCs; **a** in a female; **b** in a male)

Inter-class coefficients (ICC) were used for the analysis of reproducibility between the two observers and between the measurements taken at different times by O1. The results indicated that most ICC values were greater than 0.75. The ICCs were higher in the intra-observer comparisons (0.777–0.998 in females, 0.565–0.993 in males) than the inter-observer (0.602–0.965 in females, 0.403–0.958 in males) comparisons (Tables 3 and 4; Fig. 5).

**Table 3:** Inter-rater and intra-rater interclass correlation coefficient(ICC) in female group

	LSED		LIECD		LIED		MSED		MIECD		MIED		RSED		RIECD		RIED	
	Intra	Inter	Intra	Inter	Intra	Inter	Intra	Inter	Intra	Inter	Intra	Inter	Intra	Inter	Intra	Inter	Intra	Inter
**C3**	0.965	0.602	0.888	0.839	0.970	0.899	0.990	0.732	0.975	0.965	0.990	0.965	0.969	0.690	0.877	0.841	0.960	0.934
**C4**	0.996	0.765	0.917	0.920	0.777	0.870	0.959	0.673	0.988	0.955	0.979	0.948	0.956	0.751	0.977	0.895	0.998	0.934
**C5**	0.974	0.798	0.917	0.903	0.781	0.947	0.959	0.698	0.950	0.868	0.888	0.776	0.817	0.833	0.929	0.869	0.992	0.932
**C6**	0.995	0.839	0.883	0.778	0.997	0.937	0.976	0.698	0.929	0.943	0.962	0.952	0.992	0.847	0.979	0.829	0.996	0.935
**C7**	0.972	0.833	0.945	0.773	0.985	0.838	0.975	0.873	0.961	0.875	0.961	0.874	0.961	0.898	0.992	0.877	0.952	0.880

*LSED* depth of the superior endplate of the left para-sagittal plane, *LIECD* depth of the inferior endplate concave of the left para-sagittal plane, *LIED* depth of the superior endplate of the left para-sagittal plane, *MSED* depth of the superior endplate of the mid-sagittal plane, *MIECD* depth of the inferior endplate concave of the mid-sagittal plane, *MIED* depth of the superior endplate of the mid-sagittal plane, *RSED* depth of the superior endplate of the right para-sagittal plane, *RIECD* depth of the inferior endplate concave of the right para-sagittal plane, *RIED* depth of the superior endplate of the right para-sagittal plane

**Table 4:** Inter-rater and intra-rater interclass correlation coefficient(ICC) in female group

	LSED		LIECD		LIED		MSED		MIECD		MIED		RSED		RIECD		RIED	
	Intra	Inter	Intra	Inter	Intra	Inter	Intra	Inter	Intra	Inter	Intra	Inter	Intra	Inter	Intra	Inter	Intra	Inter
**C3**	0.894	0.782	0.872	0.802	0.863	0.808	0.579	0.403	0.768	0.928	0.746	0.958	0.923	0.663	0.565	0.839	0.766	0.930
**C4**	0.859	0.706	0.886	0.815	0.922	0.881	0.835	0.766	0.837	0.787	0.928	0.918	0.868	0.697	0.697	0.778	0.650	0.831
**C5**	0.934	0.724	0.903	0.815	0.950	0.882	0.946	0.848	0.978	0.875	0.958	0.771	0.937	0.906	0.935	0.699	0.957	0.647
**C6**	0.923	0.742	0.906	0.818	0.987	0.921	0.956	0.928	0.993	0.932	0.798	0.760	0.964	0.934	0.904	0.766	0.986	0.681
**C7**	0.966	0.852	0.993	0.774	0.978	0.892	0.959	0.932	0.883	0.658	0.982	0.930	0.974	0.864	0.905	0.929	0.931	0.806

*LSED* depth of superior endplate of left para-sagittal plane, *LIECD* depth of inferior endplate concave of left para-sagittal plane, *LIED* depth of superior endplate of left para-sagittal plane, *MSED* depth of superior endplate of mid-sagittal plane, *MIECD* depth of inferior endplate concave of mid-sagittal plane, *MIED* depth of superior endplate of mid-sagittal plane, *RSED* depth of superior endplate of right para-sagittal plane, *RIECD* depth of inferior endplate concave of right para-sagittal plane, *RIED* depth of superior endplate of right para-sagittal plane

## Discussion

### The result

The results indicated that the interclass correlation coefficients (ICCs) were excellent (> 0.75) for most occasions, and the inter-observer and intra-observer results were reliable and reproducible.

The main reason for such a result can be attributed to the fact that most landmarks we chose to establish sagittal planes had already been verified in pre-existing studies; these landmarks include the posterior endplate valley (PEV), which is a newly proposed position for landmark labeling [13] and the intersection of the bilateral laminae [14]. Both of these landmarks are stable anatomic structures as their stability guarantees the stability of the mid-sagittal plane establishment. Moreover, the midpoint of the posterior rim of the inferior endplate can be identified as a candidate landmark; the uncinate processes are stable anatomical structures located at the lateral borders of the vertebral bodies [7, 15–17]. Therefore, the mid-sagittal plane and the two following quarter para-sagittal planes can be accurately determined and established, with high reliability between observers with different occupational backgrounds as well as under different circumstances.

There are also other methods to determine the mid-sagittal plane on CT images and 3D models. The largest spinous in the sagittal plane was referred to as the mid-sagittal plane in CT data-based studies [4, 12]; for 3D models, mid-points of three lines created by connecting bilateral anatomical structures were used to generate the mid-sagittal plane [18].

### Measurement modalities

Over a long period, the most widely used and accepted method for cervical vertebrae measurement is direct measurement over the dry bone with a calliper [11, 19]. Radiographic data have been proposed, for instance [2]; however, due to the inherent disadvantages of these data, errors can be easily generated from magnification, rotation and projection, and direct measurement remains the mainstay [20, 21]. For many researchers, human bone is the preferred option rather than the ideal option, mainly due to the difficulties in obtaining and carrying bone; also, the reproducibility of the measurements in certain regions is also in doubt, especially for regions with few identifiable anatomic landmarks. The emergence of modern medical imaging modalities, such as CT and MRI, brought new considerations into endplate measurements; also, high-resolution image data have many advantages, especially with the assistance of computer programs. Gradually, CT and MRI image-based measurements have replaced the predominant position in the first decade of the twenty-first century.

Included but not limited to measurement, a digital 3D bone model, generated from CT, MRI images, and optical scanning, can serve as an alternative to replace cadaveric bone; many shortcomings of dry bone can be overcome thanks to modelling and engineering computer programs. Many in silico-based studies on 3D models have been implemented [5, 6, 8, 18] by the development of software, and studies on 3D models will increase in the future.

The 3D model has advantages, such as being relatively easier to obtain, being capable of repeated use, having fewer ethics burdens, being able to register with other image modalities, being applicable to computational simulations, and providing different authors the ability to verify the accuracy of the 3D model.

### Future prospects

The method we used in this study has the potential to be expanded into a wider area of applications. Different portions of an object can be accurately positioned at the coronal, sagittal, and axial planes for further evaluation. For example, by incorporating reference planes, measurements on cadaveric bones can also be augmented in the case of augmented reality (AR) equipment and other similar devices. Furthermore, more detailed landmarks can be generated by intersecting curves on different planes for modelling in advanced computational anatomical research.

### Limitation of the study: need further exploration

One limitation of the study is that the method has only been verified on vertebrae with no or mild degeneration, and the landmarks may be difficult to recognize in cases of severe degeneration; thus, further exploration is needed. In this study, the shape of the vertebral body is regular and has a relatively simpler topology; however, when implementing similar studies in more complex bone, such as the pelvis, or for bones in which it is difficult to define a datum reference plane, such as the humerus and femur, the method may need further adaption. Another limitation is that the samples enrolled in the study were relatively smaller, observers were limited to experienced digital medicine practitioners, and questions remain concerning further expansion to a larger population and to include observers. Additionally, measurements were carried out only on the sagittal plane, and more modifications may be considered when applied to coronal and axial planes.

## Conclusion

In this study, we propose an approach for determining the bilateral quarter para-sagittal planes on a 3D cervical vertebral body model; the reproducibility in a single observer and intra-observer reproducibility was evaluated, and the results demonstrated that the method is reproducible with high intra- and inter-observer agreement.

## Data Availability

All data generated or analyzed during this study are included in this published article.

## Abbreviations

3D: Three dimensional
CT: Computed tomography
MRI: Magnetic resonance imaging
HU: Houndsfield unit
UP: Uncinate process
ICC: Interclass correlation coefficient
PEV: Posterior endplate valley
SED: Superior endplate
IED: Inferior endplate
QPSP: Quarter para-sagittal planes
LSED: Depth of superior endplate of left para-sagittal plane
LIECD: Depth of inferior endplate concave of left para-sagittal plane
LIED: Depth of superior endplate of left para-sagittal plane
MSED: Depth of superior endplate of mid-sagittal plane
MIECD: Depth of inferior endplate concave of mid-sagittal plane
MIED: Depth of superior endplate of mid-sagittal plane
RSED: Depth of superior endplate of right para-sagittal plane
RIECD: Depth of inferior endplate concave of right para-sagittal plane
RIED: Depth of superior endplate of right para-sagittal plane
AR: Augmented reality
